# Supplementary material for: Co-expression of distinct L1 retrotransposon coiled coils can lead to their entanglement
Source: Mob DNA. 2023 Oct 20;14:16. doi: 10.1186/s13100-023-00303-8 (PMC10588031; doi:10.1186/s13100-023-00303-8)
Supplement: Supplementary file 1 — Additional file 1. [file 13100_2023_303_MOESM1_ESM.docx]

The results of control experiments shown in Panels A – D of Figure S1 support the conclusions of the main paper. Thus, HA- or FG-tagged proteins should only be present in cell extracts of HEK293F cells transfected with ORF1p-HA or ORF1p-FG expression vectors, and there should be no cross activity between their respective antibodies.

The over-exposed Western blot depicted in Panel A shows that extracts from non-transfected HEK293F cells react with neither antibody and that these cells support robust expression of HA- and FG- tagged proteins. Panels B and C show the specificity of the FG-tagged proteins (111-FG, 555-FG) for anti FG-beads, and panel D shows extracts from cells transfected with the empty pcDNA.1(+) expression vector lack any proteins that cross react with the anti-FG or anti-FA antibodies.


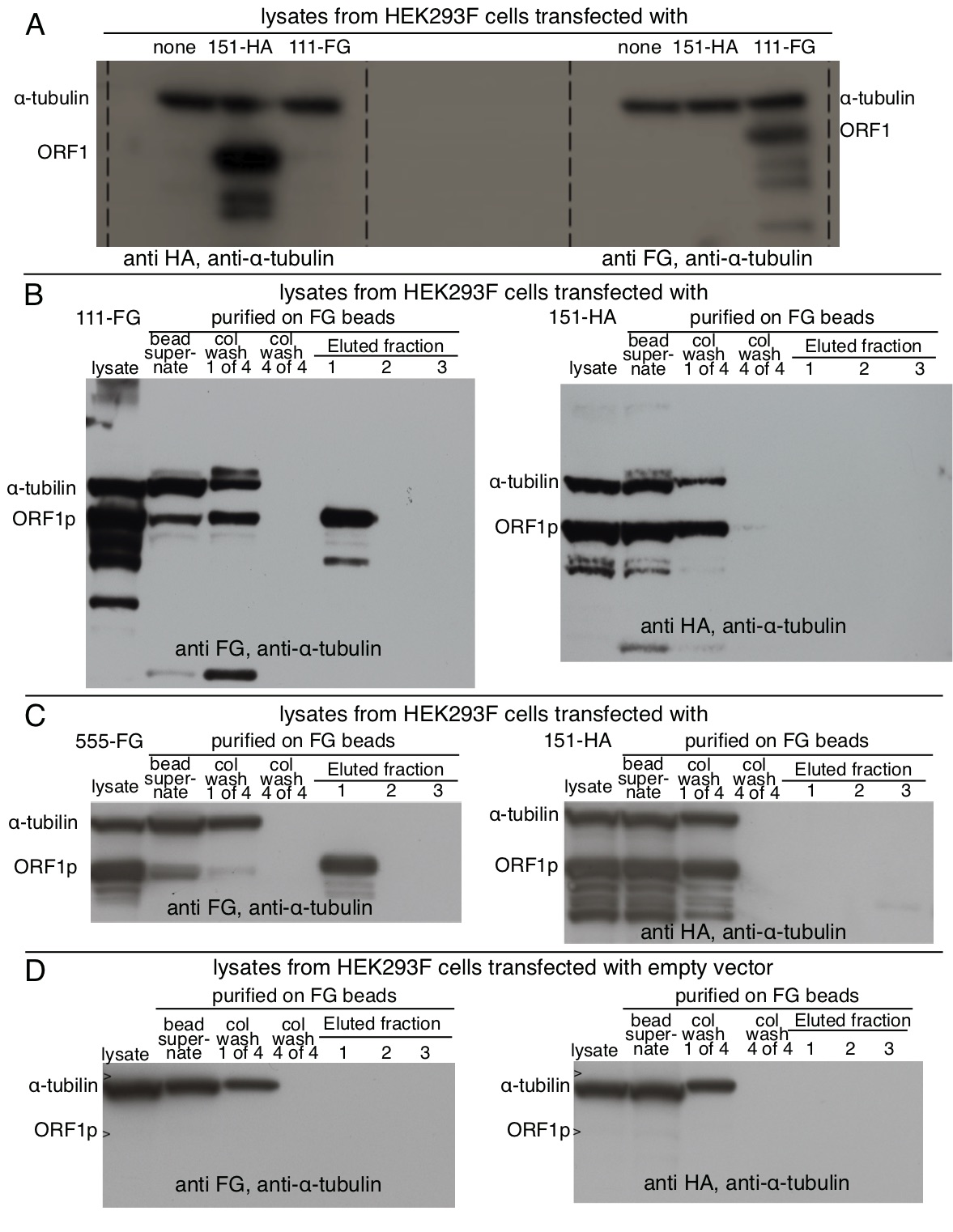


Fig. S1

Alpha tubulin has a molecular weight of 50 kDa and human ORF1p has a calculated molecular weight of 40 kDa but migrates as a ≥ 41 kDa protein on denaturing acrylamide gels: (ref. 3 – Figs 2 & 3 and ref. 29 – Fig. 1f) in the main paper.

Fig. S2

ORF1 DNA sequences (upper case) flanked by lower case 5’ BamHI and Kozak sequence compliant bases and 3’ EcoRI recognition sites. The remaining lowercase sequences encode the FG or HA epitopes.

>555-1xFG

ggatccgcaATGGGGAAAAAACAGAGCAGAAAAGCTGAAAATTCTAAAAATCAGAGCGCCTCTCCTCCTCCAAAGGAACGCAGCTCCTCACCAGCAACGGAACAAAGCTGGATGGAGAATGACTTTGACGAGTTGAGAGAAGAAGGCTTCAGACGATCAAACTTCTCCGAGCTAAAGGAGGAAGTTCGAACCCATCGCAAAGAAGCTAAAAACCTTGAAAAAAGATTAGACGAATGGCTAACTAGAATAACCAGTGTAGAGAAGTCCTTAAATGACCTGATGGAGCTGAAAACCATGGCACGAGAACTACGTGACGAATGCACAAGCTTCAGTAGCCGATTCGATCAACTGGAAGAAAGGGTATCAGTGATTGAAGATCAAATGAATGAAATGAAGCGAGAAGAGAAGTTTAGAGAAAAAAGAGTAAAAAGAAATGAACAAAGCCTCCAAGAAATATGGGACTATGTGAAAAGACCAAATCTACGTCTGATTGGTGTACCTGAAAGTGACGGGGAGAATGGAACCAAGTTGGAAAACACTCTGCAGGATATTATCCAGGAGAACTTCCCCAACCTAGCAAGGCAGGCCAACATTCAAATTCAGGAAATACAGAGAACGCCACAAAGATACTCCTCGAGAAGAGCAACTCCAAGACACATAATTGTCAGATTCACCAAAGTTGAAATGAAGGAAAAAATGTTAAGGGCAGCCAGAGAGAAAGGTCGGGTTACCCACAAAGGGAAGCCCATCAGACTAACAGCGGATCTCTCGGCAGAAACTCTACAAGCCAGAAGAGAGTGGGGGCCAATATTCAACATTCTTAAAGAAAAGAATTTTCAACCCAGAATTTCATATCCAGCCAAACTAAGCTTCATAAGTGAAGGAGAAATAAAATCCTTTACAGACAAGCAAATGCTGAGAGATTTTGTCACCACCAGGCCTGCCCTACAAGAGCTCCTGAAGGAAGCACTAAACATGGAAAGGAACAACCGGTACCAGCCACTGCAAAAACATGCCAAATTGgattataaggacgacgacgacaagtaggaattc

>111-3XFG

ggatccgcaATGGGGAAAAAACAGAACAGAAAAACTGGAAACTCTAAAACGCAGAGCGCCTCTCCTCCTCCAAAGGAACGCAGTTCCTCACCAGCAACaGAACAAAGCTGGATGGAGAATGATTTTGACGAGCTGAGAGAAGAAGGCTTCAGACGATCAAATTACTCTGAGCTACGGGAGGACATTCAAACCAAAGGCAAAGAAGTTGAAAACTTTGAAAAAAATTTAGAAGAATGTATAACTAGAATAACCAATACAGAGAAGTGCTTAAAGGAGCTGATGGAGCTGAAAACCAAGGCTCGAGAACTACGTGAAGAATGCAGAAGCCTCAGGAGCCGATGCGATCAACTGGAAGAAAGGGTATCAGCAATGGAAGATGAAATGAATGAAATGAAGCGAGAAGGGAAGTTTAGAGAAAAAAGAATAAAAAGAAATGAGCAAAGCCTCCAAGAAATATGGGACTATGTGAAAAGACCAAATCTACGTCTGATTGGTGTACCTGAAAGTGATGTGGAGAATGGAACCAAGTTGGAAAACACTCTGCAGGATATTATCCAGGAGAACTTCCCCAATCTAGCAAGGCAGGCCAACGTTCAGATTCAGGAAATACAGAGAACGCCACAAAGATACTCCTCGAGAAGAGCAACTCCAAGACACATAATTGTCAGATTCACCAAAGTTGAAATGAAGGAAAAAATGTTAAGGGCAGCCAGAGAGAAAGGTCGGGTTACCCTCAAAGGAAAGCCCATCAGACTAACAGTGGATCTCTCGGCAGAAACCCTACAAGCCAGAAGAGAGTGGGGGCCAATATTCAACATTCTTAAAGAAAAGAATTTTCAACCCAGAATTTCATATCCAGCCAAACTAAGCTTCATAAGTGAAGGAGAAATAAAATACTTTATAGACAAGCAAATGTTGAGAGATTTTGTCACCACCAGGCCTGCCCTAAAAGAGCTCCTGAAGGAAGCGCTAAACATGGAAAGGAACAACCGGTACCAGCCGCTGCAAAATCATGCCAAAATGgactacaaagaccatgacggtgattataaagatcatgacatcgattacaaggatgacgatgacaagtaggaattc

>151-HA

ggatccgcaATGGGGAAAAAACAGAACAGAAAAACTGGAAACTCTAAAACGCAGAGCGCCTCTCCTCCTCCAAAGGAACGCAGTTCCTCACCAGCAACAGAACAAAGCTGGATGGAGAATGATTTTGACGAGCTGAGAGAAGAAGGCTTCAGACGATCAAATTACTCTGAGCTACGGGAGGACATTCAAACCAAAGGCAAAGAAGTTGAAAACTTTGAAAAAAATTTAGAAGAATGTATAACTAGAATAACCAATACAGAGAAGTGCTTAAAGGAGCTGATGGAGCTGAAAACCAAGGCTCGAGAACTACGTGAAGAATGCACAAGCTTCAGCAGCCGATTCGATCAACTGGAAGAAAGGGTATCAGTTATCGAAGATCAAATGAATGAAATGAAGCGAGAAGAGAAGTTTAGAGAAAAAAGAGTAAAAAGAAATGAGCAAAGCCTCCAAGAAATATGGGACTATGTGAAAAGACCAAATCTACGTCTGATTGGTGTACCTGAAAGTGATGTGGAGAATGGAACCAAGTTGGAAAACACTCTGCAGGATATTATCCAGGAGAACTTCCCCAATCTAGCAAGGCAGGCCAACGTTCAGATTCAGGAAATACAGAGAACGCCACAAAGATACTCCTCGAGAAGAGCAACTCCAAGACACATAATTGTCAGATTCACCAAAGTTGAAATGAAGGAAAAAATGTTAAGGGCAGCCAGAGAGAAAGGTCGGGTTACCCTCAAAGGAAAGCCCATCAGACTAACAGTGGATCTCTCGGCAGAAACCCTACAAGCCAGAAGAGAGTGGGGGCCAATATTCAACATTCTTAAAGAAAAGAATTTTCAACCCAGAATTTCATATCCAGCCAAACTAAGCTTCATAAGTGAAGGAGAAATAAAATACTTTATAGACAAGCAAATGTTGAGAGATTTTGTCACCACCAGGCCTGCCCTAAAAGAGCTCCTGAAGGAAGCGCTAAACATGGAAAGGAACAACCGGTACCAGCCGCTGCAAAATCATGCCAAAATGtacccatacgatgttccagattacgcttaggaattc
